# Characterization of Exosome-Related Gene Risk Model to Evaluate the Tumor Immune Microenvironment and Predict Prognosis in Triple-Negative Breast Cancer

**DOI:** 10.3389/fimmu.2021.736030

**Published:** 2021-10-01

**Authors:** Pengjun Qiu, Qiaonan Guo, Qingzhi Yao, Jianpeng Chen, Jianqing Lin

**Affiliations:** Department of Breast and Thyroid Surgery, The Second Affiliated Hospital of Fujian Medical University, Quanzhou, China

**Keywords:** TNBC, exosome, immune cell infiltration, risk model, ESTIMATE

## Abstract

**Background:**

As a kind of small membrane vesicles, exosomes are secreted by most cell types from multivesicular endosomes, including tumor cells. The relationship between exosomes and immune response plays a vital role in the occurrence and development of tumors. Nevertheless, the interaction between exosomes and the microenvironment of tumors remains unclear. Therefore, we set out to study the influence of exosomes on the triple-negative breast cancer (TNBC) microenvironment.

**Method:**

One hundred twenty-one exosome-related genes were downloaded from ExoBCD database, and IVL, CXCL13, and AP2S1 were final selected because of the association with TNBC prognosis. Based on the sum of the expression levels of these three genes, provided by The Cancer Genome Atlas (TCGA), and the regression coefficients, an exosome risk score model was established. With the median risk score value, the patients in the two databases were divided into high- and low-risk groups. R clusterProfiler package was employed to compare the different enrichment ways between the two groups. The ESTIMATE and CIBERSORT methods were employed to analyze ESTIMATE Score and immune cell infiltration. Finally, the correlation between the immune checkpoint-related gene expression levels and exosome-related risk was analyzed. The relationship between selected gene expression and drug sensitivity was also detected.

**Results:**

Different risk groups exhibited distinct result of TNBC prognosis, with a higher survival rate in the low-risk group than in the high-risk group. The two groups were enriched by immune response and biological process pathways. A better overall survival (OS) was demonstrated in patients with high scores of immune and ESTIMATE rather than ones with low scores. Subsequently, we found that CD4^+^-activated memory T cells and M1 macrophages were both upregulated in the low-risk group, whereas M2 macrophages and activated mast cell were downregulated in the low-risk group in patients from the TCGA and GEO databases, respectively. Eventually, four genes previously proposed to be targets of immune checkpoint inhibitors were evaluated, resulting in the expression levels of CD274, CTLA4, LAG3, and TIM3 being higher in the low-risk group than high-risk group.

**Conclusion:**

The results of our study suggest that exosome-related risk model was related to the prognosis and ratio of immune cell infiltration in patients with TNBC. This discovery may make contributions to improve immunotherapy for TNBC.

## Introduction

Breast cancer (BC) is the most prevalent type of cancer and the most common cause of cancer-related deaths among women worldwide (1). TNBC is a subtype of breast cancer histologically defined by the lack of estrogen receptor (ER), progesterone receptor (PR), and HER-2 overexpression (2). Although this subtype of BC accounts for approximately 15%–20% of BC cases worldwide, it is associated with higher incidence of local recurrence and metastasis. In the last decades, the treatment of TNBC has been limited to surgery, chemotherapy, and radiotherapy (3). More recently, biomarker-driven therapies and immune checkpoint inhibitors are demonstrated as promising selections for a subset of TNBC treatment (4). Additionally, as a class of potent anticancer drugs, antibody-drug conjugates are approved for TNBC by FDA (5). Nevertheless, numbers of TNBC patients experience disease progression (DP) within 2–5 years from initial diagnosis (6). Hence, better understanding of the underlying mechanisms involved in TNBC progression and effective treatments against TNBC are urgently needed.

As a type of extracellular vesicles (EVs), exosomes are homogenous membrane vesicles (ranging from 30 to 150 nm), derived from the multivesicular bodies (MVBs), formed by the budding of the endosomal membranes and released in the extracellular space upon fusion with the plasma membrane (7, 8). In 1983, exosomes were discovered and considered to operate as cellular garbage disposal (9, 10). With the investigation of biological function, angiogenesis, immunity, and metastasis have been demonstrated to be regulated by exosomes from cancer cells, making a critical effect in facilitating tumorigenesis (8, 11, 12). Interestingly, some studies indicated that one part of the human epidermal growth factor receptor (HER) family was exosome associated in breast, gastric, and pancreatic cancers (8, 13–15). Moreover, other studies identified higher expression levels of serum exosomal-annexin A2 (exo-AnxA2) in female with breast cancer against noncancer, especially for TNBC rather than luminal and HER2-positive BC (16). Consequently, further searches for exosomes in breast cancer are emerging as a highly potential method for diagnosis and treatment of breast cancer.

In 1996, immunologists discovered that Epstein-Barr virus-transformed B lymphocytes were able to secrete exosomes *via* fusion of MVBs with the plasma membrane (17).

On the other hand, exosomes released by some tumors also contain a variety of immunosuppressive molecules (18), which can reduce proliferation of CD4 and CD8 T lymphocytes (19–22) or natural killer (NK) cells (23, 24) or promote the differentiation of regulatory T lymphocytes, myeloid cells, and immunosuppressive cells *in vitro* (25, 26). Consequently, exosomes are closely related to immunotherapy of malignant tumor. Recently, immunotherapy has made appreciable progress in antitumor practice. Based on the immune regulation between the tumor microenvironment (TME) and cancer cells, the clinical benefits of immunotherapy are achieved compared with the traditional treatments by stimulating a sustained antitumor immune response (27). As a vital part of the TME, infiltrating immune cells are considered closely related to tumor progression and the immunotherapy efficacy (28, 29). Previous studies in early stage TNBC and HER2-positive BC suggested potential benefit of immune activation to improve prognosis (30). Immune checkpoint blockade monotherapy and immunotherapy in combination with chemotherapy have been reported to achieve encouraging results in treatment of some subtypes of breast cancer (31, 32). Hence, it is critical to identify novel biomarkers to identify immunotherapy responsive subtypes of breast cancer.

This study aimed to explore the relationship between exosome-associated genes and the immune microenvironment of TNBC. In the present study, the gene profile data of TNBC patients were extracted from the TCGA and GEO databases and genetic data related to exosomes were downloaded from ExoBCD database to analyze and construct an exosome risk model. The prognostic prediction for TNBC patients was conducted according to this risk model. Subsequently, the exosome risk score was used as the entry point to investigate distinction in the infiltration rate of immune cells. On this basis, the interrelation between exosome risk score and tumor immune microenvironment was further searched and four genes (33–35) previously reported associated to immune checkpoint inhibitors were also analyzed. In the future, this crucial treatment method will be employed to develop numbers of interesting combination therapy strategies.

## Materials and Methods

### Data Acquisition

Clinical information and RNA-sequencing expression date of TNBC patients were collected from the TCGA (http://cancergenome.nih.gov/) as a training set. Subsequently, the corresponding information of TNBC patients from the GSE58812 dataset was downloaded from the GEO database (https://www.ncbi.nlm.nih.gov/geo/) as a validation set. A total of 1,066 TNBC samples from TCGA and 107 TNBC samples from the GEO were included in our study. Followed by batch normalization, the patients were removed due to clinical data being incomplete and overall survival was less than 90 days. Thus, 123 TNBC samples from the TCGA and 105 TNBC samples from the GEO with complete follow-up information were enrolled in our training data set and validation set for further analyses, respectively. Afterwards, 121 exosome-related genes were downloaded from ExoBCD database (https://exobcd.liumwei.org/) and provided in **Supplementary S1**.

### Constitution of a Risk Model

The genes related to exosome in the TCGA cohort were ascertained by Venn diagram. Univariate Cox analysis of overall survival (OS) was performed to select exosome-related genes with independent prognostic significance and visualized *via* forest plots. Afterwards, the least absolute shrinkage and selection operator (LASSO) Cox regression model was applied to reduce redundant genes and obviate model overfitting. Accordingly, all independent prognostic genes were determined in the model (36). The risk score of patients was calculated according to the gene expression level and the risk score formula was constructed as: $\text{Risk Score}=\sum_{i=1}^{n}(\text{Ex}{\text{p}}_{i}*\text{Co}{\text{e}}_{i})$ . (*N* = 3, Exp*_i_* indicated the expression level for each exosome-related genes, and Coe*_i_* indicated the corresponding Cox regression coefficient.) Afterwards, patients were divided into high- and low-risk groups based on the median risk score of the TCGA cohort. According to the signature of genes expression, PCA was conducted by the “prcomp” function of the “stats” R package. Survival analysis between the different risk groups were performed with the “survminer” R package. The predictive accuracy of the gene signature was assessed by time-dependent ROC curve analysis. Consequently, the risk score was demonstrated as an independent prognostic factor for TNBC patients by univariate and multivariate COX regression analysis. Bilateral *p* < 0.05 were considered statistically significant, and the 95% confidence intervals were determined by calculating the hazard ratio (HR).

### Functional Enrichment Analysis

The functional enrichment analyses including Gene Ontology (GO) enrichment and Kyoto Encyclopedia of Genes and Genomes (KEGG) pathway analyses were carried out for the different expression genes (DEGs) between high- and low-risk cohorts by means of the R clusterProfiler package. Also, biological process (BP), molecular function (MF), and cellular component (CC) are included in GO terms. GO terms and KEGG pathways with *p*-values <0.05 were considered significant.

### Evaluation of Tumor Microenvironment in TNBC Cohorts

Estimation of stromal and immune cells in malignant tumor tissues using expression (ESTIMATE) algorithm was adopted to calculate the ratio of the immune-stromal component in TME through “estimate” R package, which generates immune score, stromal score, and ESTIMATE score (37). Subsequently, the immune score, stromal score, and ESTIMATE score were calculated in the high- and low-risk groups, respectively. The higher the respective score, the greater is the proportion of the corresponding component in TME.

### Evaluation of Immune Cell Type Components

CIBERSORT (http://cibersort.stanford.edu/) is a common method for immune cell infiltration estimation and analysis, evaluating the ratio of diverse cell subtypes in mixed cell samples by RNA-seq expression profile. Naive and memory B cells, seven types of T cells, NK cells, plasma cells, and myeloid subsets are all included in 22 marked immune cell subtypes, of which, the annotated gene expression signatures are visualized by LM22. CIBERSORT was used to evaluate the proportion of 22 immune cell subtypes in the high- and low-risk groups (38). The hypothesis of the type of immune cells was considered accurate and statistically significant for further analysis with the *p* < 0.05. Hence, the CIBERSORT algorithm was employed to assess the fractions of tumor immune infiltrating cell (TIIC) type components in each TNBC sample. The Wilcoxon’s test was conducted to distinguish the characteristics of TIIC between high- and low-risk group tissues.

### Correlations Between Gene Expression and Exosome-Related Risk

The genes playing a critical role in immune cell regulation was selected. Subsequently, the ggplot2, GGPUBR, and ggExtra packages in R were employed to identify the relationships between gene expression levels and different risks of exosomes.

### Drug Sensitivity Analysis

CELLMINER website (https://discover.nci.nih.gov/cellminer/) is a tool for NCI-60 database analysis, including 60 cancer cell line information. The mRNA profiles and drug sensitivity IC_50_ values of the NCI-60 panel of human cancer cell lines were extracted from the website, and then, the therapeutic effects of 163 Food and Drug Administration (FDA)-approved drugs in TNBC patients were determined. The Wilcoxon’s test was employed to analyze the significance between differences in the IC_50_ Z-score between the high- and low-risk groups. Results were presented in terms of box drawings plotted by the ggplot2 function of R.

### Statistical Analysis

All statistical analyses were conducted by R software (version 4.0.5) (https://www.r-project.org/). The association of clinicopathological variables in TNBC patients between high- and low-risk cohorts was subjected to the Chi-square test. The differences between variables of two groups were examined by using the Wilcoxon’s test. Kaplan-Meier curve was employed to assess the survival data. Independent prognostic factors were evaluated *via* univariate and multivariate Cox regression analyses. *p* < 0.05 was considered statistically significant.

## Results

### Characterization of Exosome Risk Score to Predict TNBC Prognosis

The basic characteristics of TNBC patients in the TCGA database and the GEO database are presented in **Table 1**.

**Table 1:** Clinicopathological characteristics of TNBC patients in this study.

Risk	TCGA (*n* = 123)								GEO (*n* = 105)			
	Survival		Age		Stage		Race		Survival		Age	
	Alive	Dead	≤60	>60	I–II	III–IV	White	Other	Alive	Dead	≤60	>60
**High**	47	14	45	16	46	15	34	27	40	23	34	29
**Low**	59	3	41	21	54	8	38	24	36	6	29	13
***p***	<0.01		0.467		0.152		0.658		<0.023		0.178	

**Figure 1:**
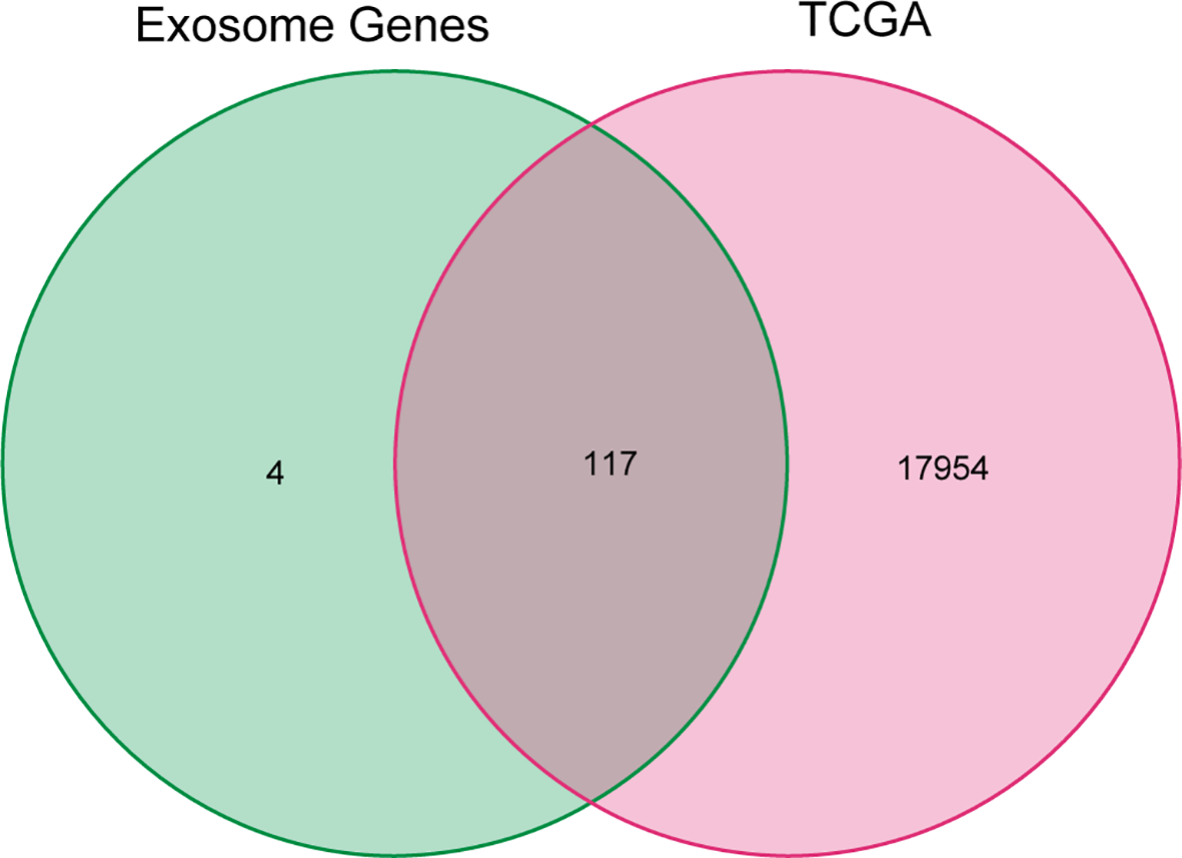
Venn diagram of genes in TCGA cohort and exosome-related genes. The 121 exosome-associated genes were downloaded from ExoBCD database, of which, 117 genes related to exosome in the TCGA cohort were ascertained by Venn diagram.

The exosome-associated gene set from ExoBCD database was downloaded, which contains 121 genes involved in immune regulation pathways. One hundred seventeen genes related to exosome in the TCGA cohort were ascertained by Venn diagram (**Figure 1**).

**Figure 2:**
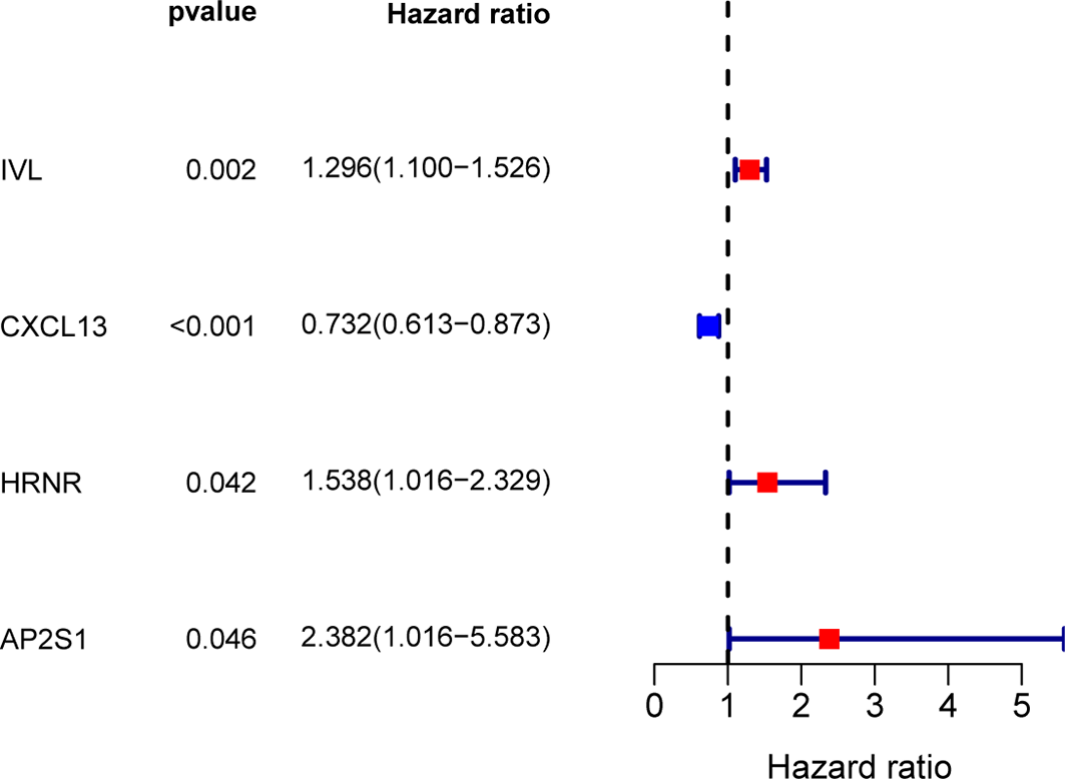
Screening for exosome-related genes associated with prognosis of TNBC patients by univariate Cox regression analysis. Four exosome-related genes were confirmed to be associated with patient’s OS (*p* < 0.05).

**Figure 3:**
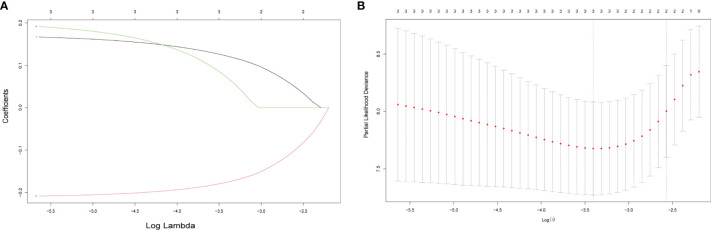
Selecting exosome-related genes associated with patient prognosis by LASSO Cox regression analysis. **(A)** LASSO coefficient profiles of three genes with *p* < 0.01. **(B)** Tenfold cross-validations result which identified optimal values of the penalty parameter *λ*.

To build an exosome risk model to predict the prognosis of TNBC patients, univariate Cox regression analysis was carried out for initial screening on 117 common genes in the TCGA training dataset. Hence, the interferences form excessive confounding genes were removed and the genes with the most significant effect on prognosis were selected. After the univariate Cox regression analysis, four exosome-related genes were confirmed to be associated with patient’ OS (*p* < 0.05). The relationship between each gene and overall survival was visualized by forest plot (**Figure 2**). To avoid exclusion of important prognosis genes, four genes mentioned were moved into LASSO regression and three significant independent prognostic genes were identified. Subsequently, the LASSO coefficient profiles of the three genes were presented (**Figure 3A**) and 10-fold crossvalidation results were produced to determine optimal values of the penalty parameter *λ* (*λ* = 0.03317128) (**Figure 3B**).

According to these results, three-gene prognostic model to assess the OS of TNBC patients was constructed according to the expression of the three genes and their regression coefficients as follows: Risk score = (0.122 × expression level of IVL) + (−0.176 × expression level of CXCL13) + (0.072 × expression level of AP2S1). Subsequently, the median risk score of TCGA cohort was set as the cutoff value to separate the patients into low- and high-risk groups.

**Figure 4:**
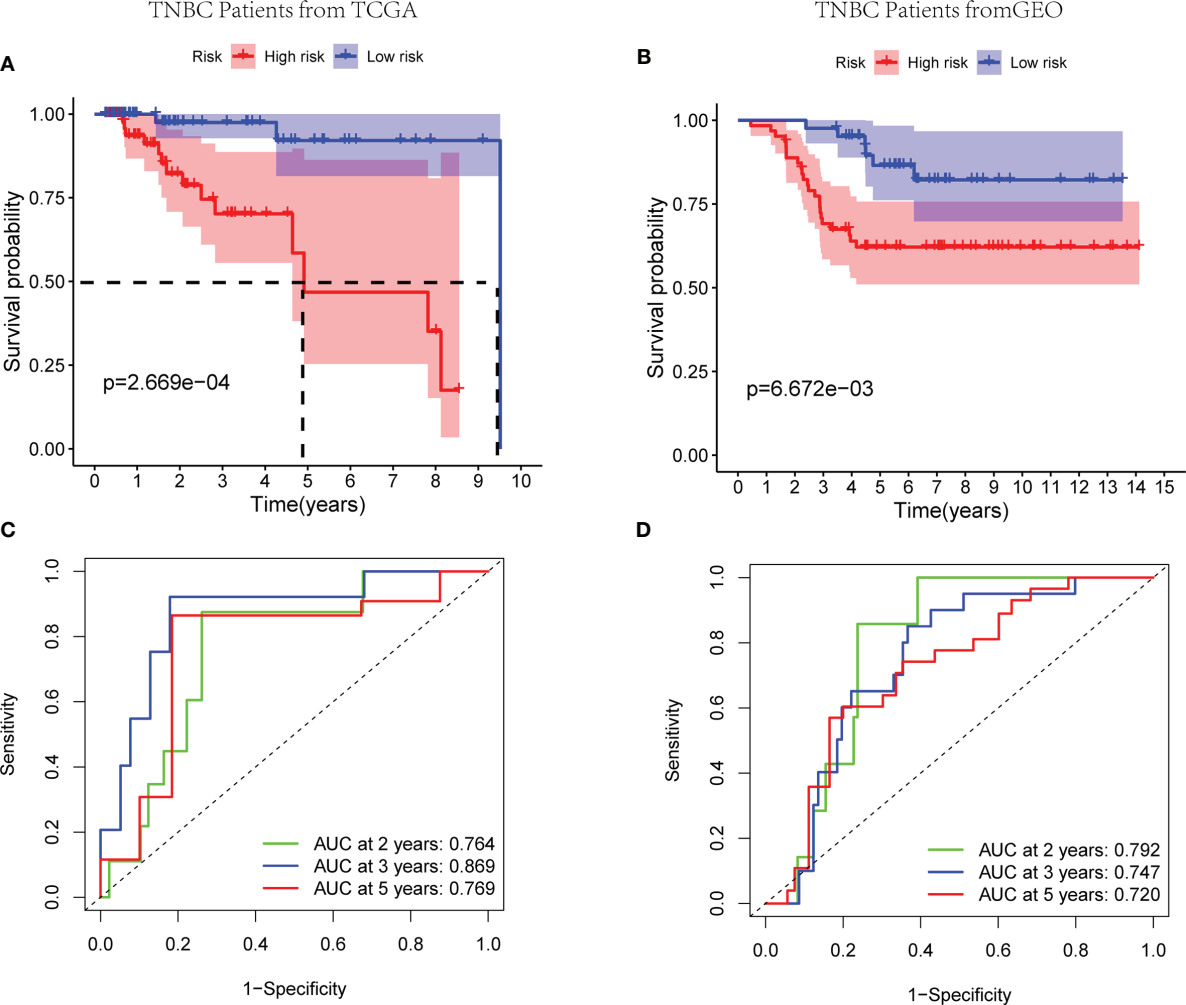
Efficacy of the exosome risk model on prognosis of TNBC patients. **(A, B)** Kaplan-Meier survival curves for TNBC patients from TCGA and GEO databases, stratified according to risk scores (high *vs.* low). **(A)** The median survival time in high- and low-risk groups of TCGA cohort are 4.91 and 9.51 years, respectively; comparisons of the survival time in high- and low-risk groups with log-rank tests (*p* = 2.67*E*−04); the hazard ratio of TCGA cohort is 6.25; the 95% CI for high- and low-risk groups are presented as the red and blue shaded parts, respectively. **(B)** Patients in both the high- and low-risk groups of GEO cohort have not achieved median survival times; comparisons of the survival time in high- and low-risk groups with log-rank tests (*p* = 6.67*E*−03); the hazard ratio of GEO cohort is 3.24; the 95% CI for high- and low-risk groups are presented as the red and blue shaded parts, respectively. **(C, D)** ROC curve analysis of the accuracy of the model to predict patient prognosis at 2, 3, and 5 years in the training set **(C)** and the validation set **(D)**.

Worse survival rates were indicated in patients with high-risk score in the training set by the Kaplan-Meier curves (*p* < 0.01) (**Figure 4A**). Afterwards, a time-dependent ROC analysis was carried out at 2, 3, and 5 years to evaluate the prognostic accuracy of the risk score. Therefore, the identified prognostic signature had been validated to be robust efficient by the area under the curve (AUC) in predicting the OS for TNBC patients (AUC = 0.764, 0.869, and 0.769 at 2, 3, and 5 years, respectively, **Figure 4C**). Analogously, 105 patients from GSE58812 were selected as the validation cohort, and the risk score of every patient was calculated according to mentioned three-gene signature. Subsequently, the median risk score of TCGA cohort was set as the cutoff value to separate the patients in the validation cohort into low- and high- risk groups. Based on the Kaplan-Meier curves (*p* < 0.01), the worse outcomes were observed in high-risk patients (**Figure 4B**). Notably, the risk score had been verified with a good long-term prognostic accuracy, shown in the time-dependent ROC analysis (AUC = 0.792, 0.747, and 0.720 at 2, 3, and 5 years, respectively, **Figure 4D**). Therefore, a three-gene signature to assess the prognosis of TNBC patient was established successfully.

**Figure 5:**
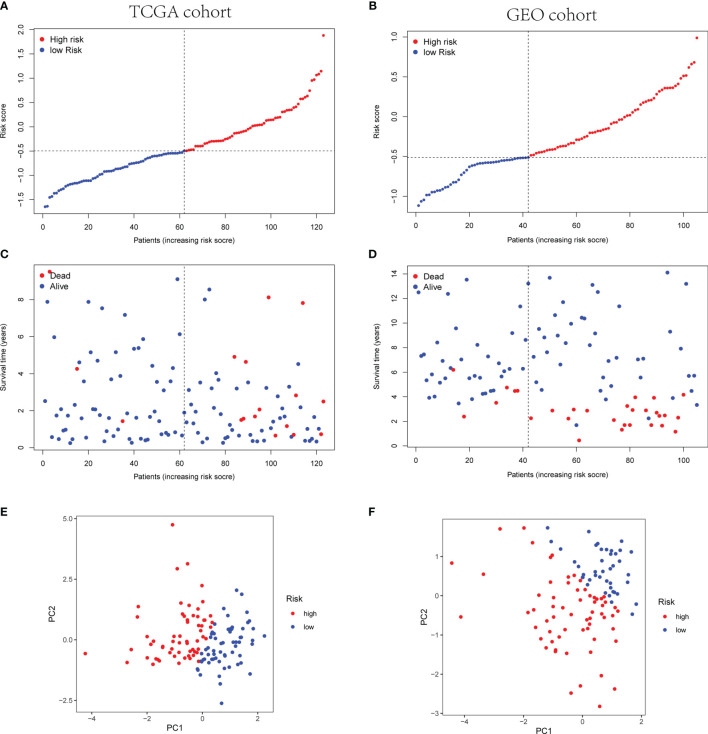
Prognostic analysis of the three-gene signature model in TCGA cohort and GEO cohort. **(A, B)** The distribution and median value of the risk scores in TCGA cohort **(A)** and the median risk score of TCGA was set as the cutoff value of high- and low-risk groups in GEO cohort **(B)**. **(C, D)** The distributions of OS status and risk scores in TCGA cohort **(C)** and GEO cohort **(D)**. **(E, F)** PCA analysis plot of TCGA cohort **(E)** and GEO cohort **(F)**.

Afterwards, according to the median risk score of the TCGA cohort, the TNBC patients from the TCGA and GEO cohorts were divided into high- or low-risk group (**Figures 5A, B** **)**. The high-risk group showed poor prognosis rather than the low-risk group (**Figures 5C, D** **)**. Principal component analysis (PCA) indicated the patients were categorized in opposed directions according to the distinct risk groups. The results of PCA were similar between the GEO cohort and TCGA cohort (**Figures 5E, F** **)**.

**Figure 6:**
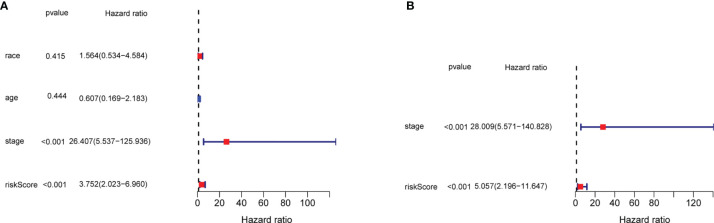
The results of the univariate and multivariate Cox regression analyses regarding significant survival-related clinical characteristic parameters in TCGA. **(A)** The forest plots for univariate Cox regression analysis show that risk score (high risk vs. low risk) and AJCC stage (stages I and II *vs.* III and IV) were prognostic risk-related variables. **(B)** The forest plots for multivariate Cox regression analysis show risk score (high risk *vs.* low risk) and AJCC stage (stages I and II *vs.* III and IV) were independent prognostic factors.

In order to evaluate the efficacy of the three-gene signature to be an independent predictor of prognosis for TNBC patients, the three-gene signature along with covariates including race, age, and tumor stage were brought into the univariate and multivariate Cox regression analysis. The univariate Cox regression analysis revealed that the tumor stage and exosome-related risk score were independent variables for forecasting the prognosis of TNBC patients in the TCGA cohort (*p* < 0.001, HR = 26.407 (95% CI, 5.537–125.936) and *p* < 0.001, HR = 3.752 (95% CI, 2.023–6.960), *p* < 0.001, **Figure 6A**). The multivariate Cox regression analysis also demonstrated that the stage and exosome-related risk score were independent prognostic factors of TNBC patients in the TCGA cohort (*p* < 0.001, HR = 28.009 (95% CI, 5.571–140.828) and *p* < 0.001, HR = 5.057 (95% CI, 2.196–11.647), *p* < 0.001, **Figure 6B**).

### KEGG and GO Functional Enrichment Analysis

**Figure 7:**
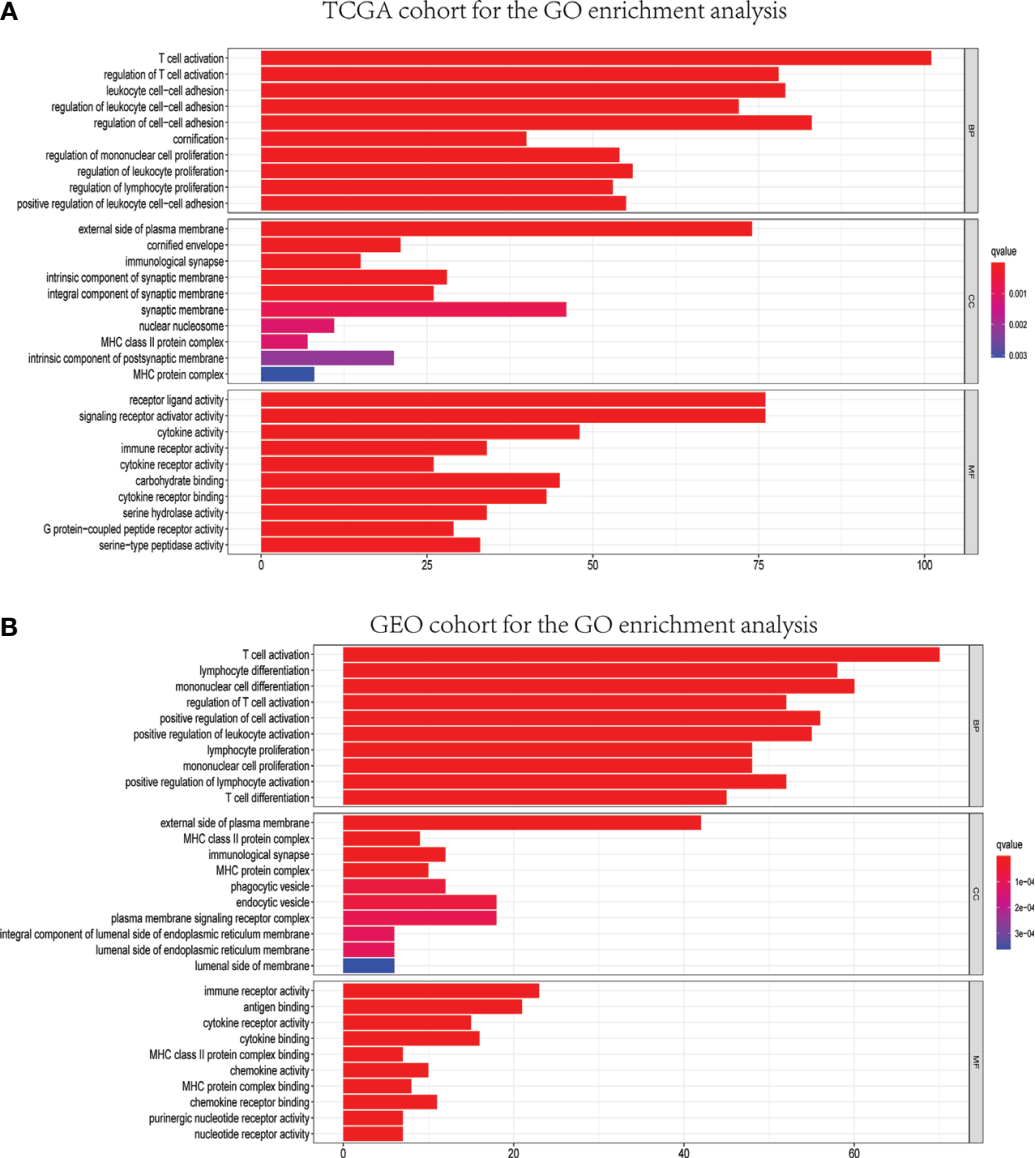
Representative results of GO enrichment analysis in TCGA and GEO databases. The results of GO biological process enrichment, GO cellular component enrichment, and GO molecular function enrichment of DEGs between different risk groups in TCGA **(A)** and GEO **(B)** cohorts.

**Figure 8:**
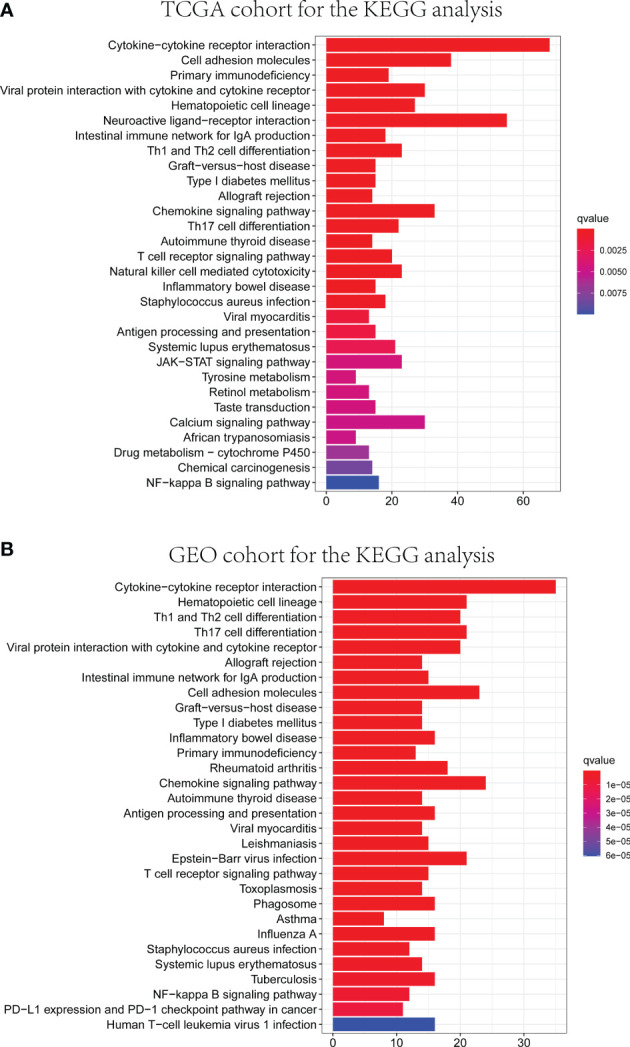
Representative results of KEGG enrichment analysis in TCGA and GEO databases. The results of KEGG pathway analysis of DEGs between different risk groups in TCGA **(A)** and GEO **(B)** cohorts.

GO and KEGG analyses were conducted to clarify the biological functions and pathways related to the risk score. The top 30 enriched GO terms are manifested in **Figure 7**, based on biological process (BP), cellular component (CC), and molecular function (MF). Also, the 30 enriched KEGG pathways are manifested in **Figure 8**, as well. Among them, a majority of GO terms and KEGG pathways were related to immune responses and biological processes.

### Correlation of ESTIMATE Score and Exosome-Related Gene Prognostic Model

**Figure 9:**
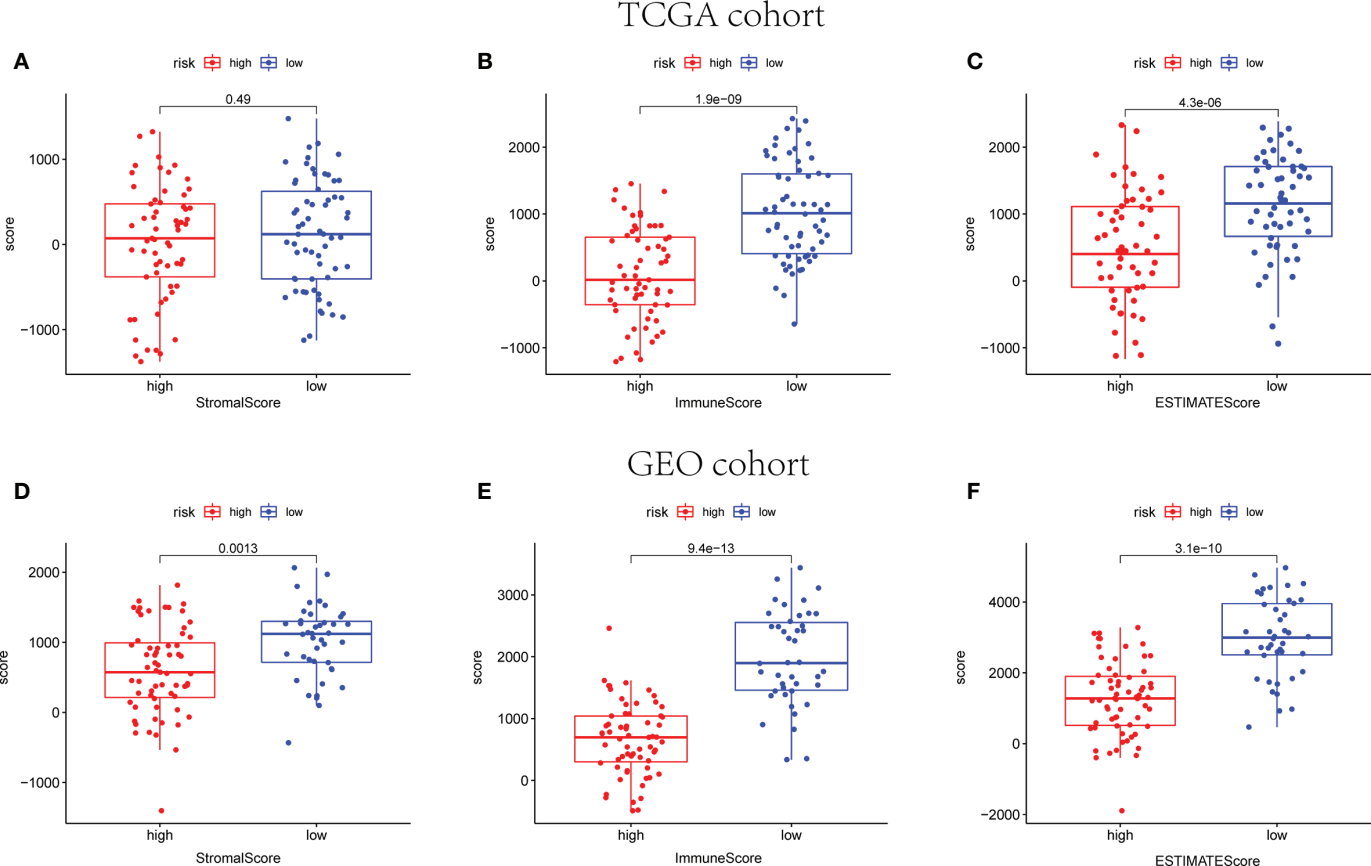
The scatter plot shows that the stromal scores, the immune scores, and the ESTIMATE scores are distributed differently between different risk groups in TCGA **(A–C)** and GEO cohorts **(D–F)**.

The ESTIMATE algorithm was employed to calculate the ESTIMATE score of every sample, reflecting the TME landscape and the overall degree of immune infiltration (37). As shown in **Figure 9**, both in training and validation cohorts, patients in the low-risk group are proved with higher immune and ESTIMATE scores than patients in the high-risk group (*p* < 0.05).

### Infiltrating Immune Cell Distribution in TNBC

**Figure 10:**
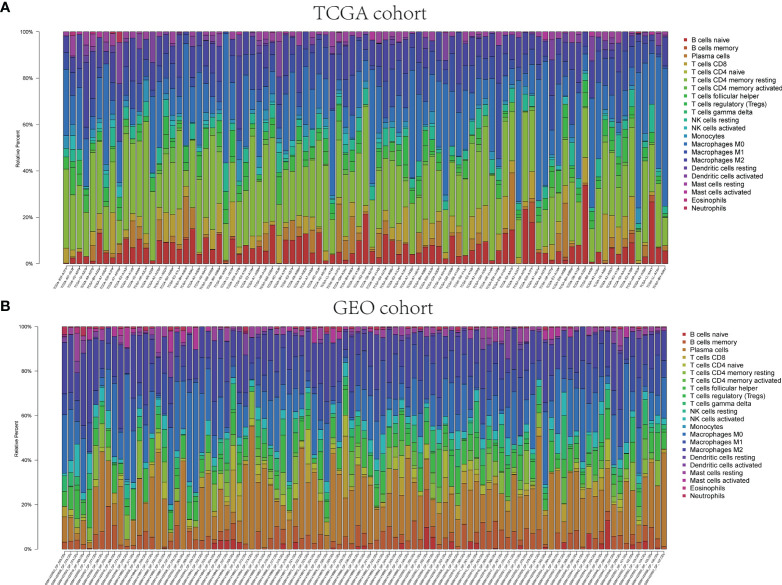
Immune infiltrations of TCGA and GEO cohorts. Relative proportion of immune infiltration in TCGA **(A)** and GEO **(B)**.

**Figure 11:**
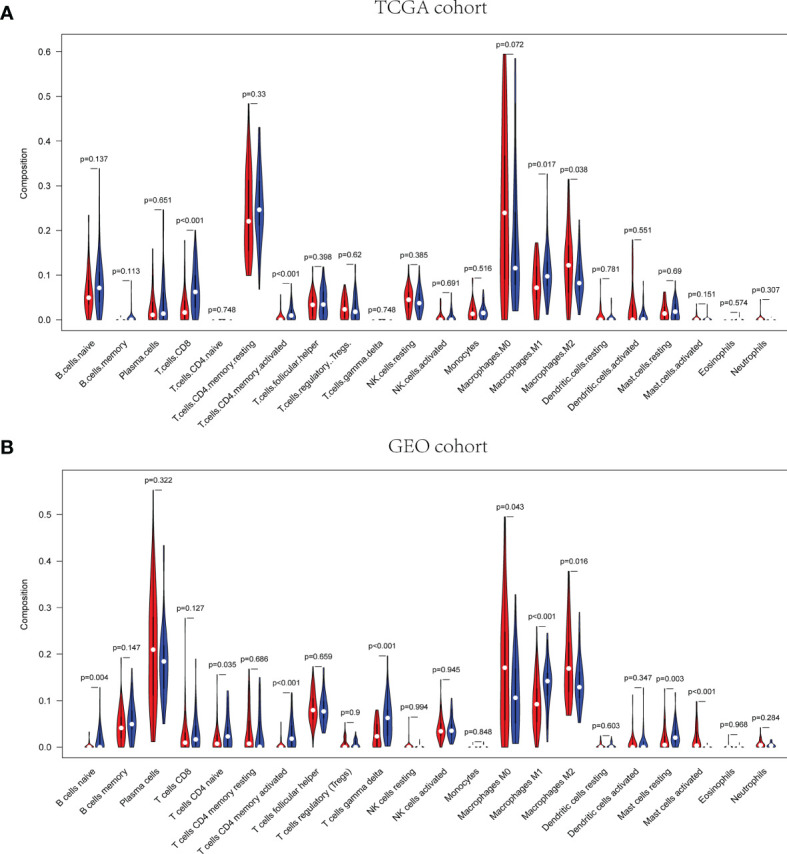
Correlation of distinct different immune cells between high- and low-risk groups in TCGA **(A)** and GEO **(B)**, respectively.

The pathway enrichment analysis indicated that the DEGs of exosome-related genes between high- and low-risk groups commonly enriched in the pathways associated to immunity, inflammation, and so on. Accordingly, CIBERSORT algorithm was employed to calculate TIIC proportions and establish 22 kinds of TIIC profiles. **Figures 10A, B** shows the ratio of immune cell infiltration in TCGA and GEO databases, respectively. As shown in **Figures 11A, B**, CD8^+^ T cells (*p* < 0.001), CD4^+^-activated memory T cells (*p* < 0.001), and M1 macrophages (*p* = 0.017) were upregulated, while M2 macrophages (*p* = 0.038) was downregulated in the low-risk group in patients from TCGA. As for GEO database, naïve B cells (*p* = 0.004), CD4^+^ naïve T cells (*p* = 0.035), CD4^+^-activated memory T cells (*p* < 0.001), gamma delta T cell (*p* < 0.001), M1 macrophages (*p* < 0.001), and resting mast cell (*p* = 0.003) were upregulated, while M0 macrophages (*p* = 0.043), M2 macrophages (*p* = 0.016), and activated mast cell (*p* < 0.001) were downregulated in the low-risk group. Hence, targeting exosome-related gene research can be a seminal discovery for future immunotherapy for tumor patients.

### Immune Checkpoint Gene Expression in Each Risk Group

**Figure 12:**
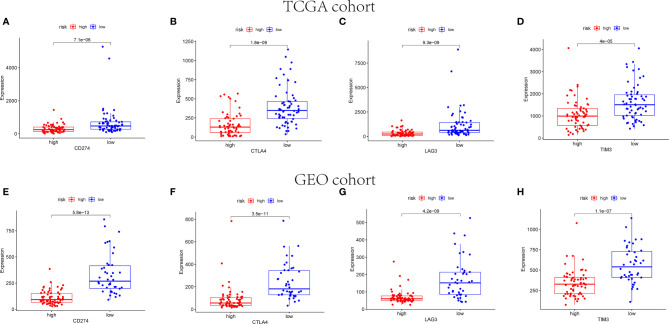
Immune checkpoint gene expression levels in high- and low-risk groups. The expression levels of CD274 **(A)**, CTLA4 **(B)**, LAG3 **(C)**, and TIM3 **(D)** in each group in TCGA cohort (*p* < 0.001). The expression levels of CD274 **(E)**, CTLA4 **(F)**, LAG3 **(G)**, and TIM3 **(H)** in each group in GEO cohort (*p* < 0.001).

The expression of immune checkpoint genes related to the treatment response of immune checkpoint inhibitors was also analyzed. The expression status of four genes formerly raised to be targets of immune checkpoint inhibitors were evaluated: PD-L1(CD274), CTLA4, LAG3, and TIM3. **Figure 12** reveals that the expression levels of CD274, CTLA4, LAG3, and TIM3 were higher in the low-risk group than the high-risk group.

### Drug Sensitivity Analysis for Independent Prognostic Genes Related to Exosomes

**Figure 13:**
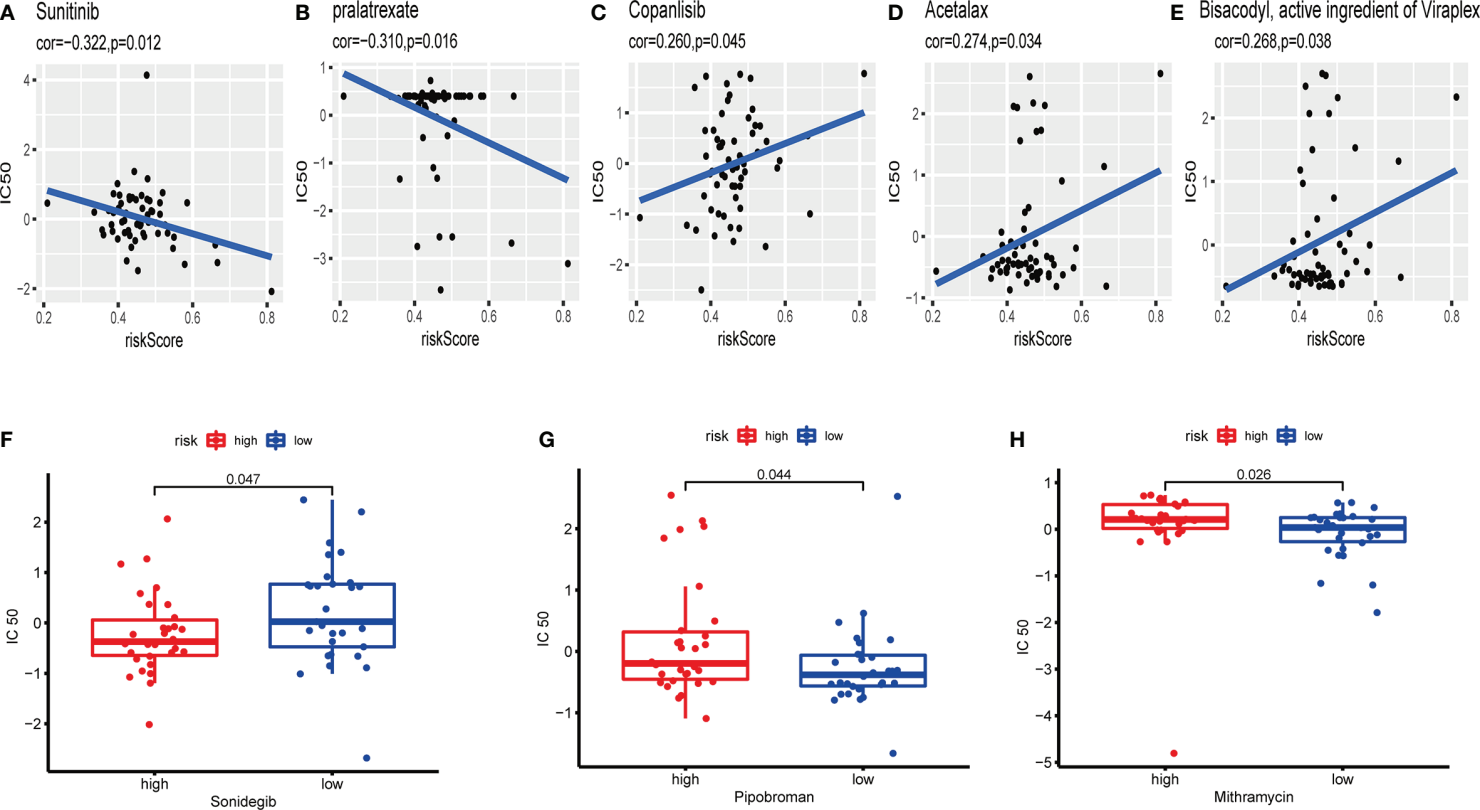
The exosome-related risk model as a potential predictor for chemosensitivity. **(A–E)** The respective IC_50_ value of chosen compounds in relation to the risk score, as shown by Pearson’s correlation analysis (sunitinib, pralatrexate, copanlisib, acetalax, and bisacodyl) appeared to associate significantly with the exosome-related risk model (|Pearson’s correlation|>0.25 and *p* < 0.05). **(F)** Those with high-risk scores were found to possess lower IC_50_ scores for FDA-approved chemotherapeutics such as sonidegib (*p* < 0.05). **(G**, **H)** Those with low-risk scores were indicated to possess lower IC_50_ scores for medications including pipobroman and mithramycin (*p* < 0.05). The *p*-values were calculated using the Wilcoxon’s test.

CellMiner database was adopted to evaluate the significance between the distinctions between the high- and low-risk cohorts on drug sensitivity for better precision treatment. Z-score is a tool to measure the drug sensitivity, and the higher the score, the more sensitive to the drug treatment. The exosome-related risk score of NCI60 cell lines was calculated, and the relationship between the risk score and the inhibitory centration (IC_50_) value of 163 FDA-approved drugs across 60 cell lines were further analyzed. As consequence, sunitinib, pralatrexate, copanlisib, acetalax, and bisacodyl appeared to associate significantly with the exosome-related risk model (|Pearson’s correlation|>0.25 and *p* < 0.05, **Figures 13A–E**). Notably, a high-risk score was linked to a lower half IC_50_ of sonidegib (Wilcoxon’s test, *p* = 0.047, **Figure 13F**). However, a low-risk score was related to a lower half IC_50_ of medications including pipobroman (Wilcoxon’s test, *p* = 0.044, **Figure 13G**) and mithramycin (Wilcoxon’s test, *p* = 0.026, **Figure 13H**). These findings indicated that the model was probable to function as a chemosensitivity predictor.

## Discussion

In a majority of cell types, multivesicular endosomes release small membrane, such as exosomes, playing vital roles in cell-to-cell communications (18). Exosomes are investigated in various biological functions, including antigen presentation, immune regulation, apoptosis evasion, drug resistance, immune surveillance escape, and so on (8, 18). In addition, exosomes secreted from some malignant tumor cells were considered a key in regulating angiogenesis, immunity, and metastasis to promote tumorigenesis (11, 12). Some studies reported that exosomes were easily available and stable *in vitro*. Therefore, researchers suggested that exosomes would have huge potentiality in malignant tumor diagnosis and treatment in early stage (39). By way of example, the finds in pancreatic ductal adenocarcinomas by Bruno Costa-Silva et al. suggested that exosomal macrophage migration inhibitory factor (MIF) primed the liver for metastasis because of the high expression of MIF in PDAC-derived exosomes and the blockade of MIF could prevent liver premetastatic and metastatic niche formation (40). The study in melanoma by Peinado et al. found that the metastatic behavior of primary tumors was governed by the transfer of exosomes to bone marrow progenitor cells *via* MET receptor (11). Additionally, the exosome-related liquid biopsy approach has been applied to detect prostate and lung cancer markers (41, 42). Wang et al. discovered that exosomal tetraspanin CD82 was associated with BC progression and the high expression levels of CD82 were detected in BC patient serum (43). More recently, automated micro flow cytometer was employed by Kibria et al. to analyze expression status of exosomes isolated from tumor cells and blood of BC patients (44). A significant reduction in CD47 expression in circulating exosomes was observed in breast cancer patients (45). Furthermore, some studies in breast cancer indicate that exosomal proteins and microRNAs may also be used as cancer biomarkers. Gonzalez Villasana et al. confirmed the higher concentrations of exosomes in breast cancer patients by isolating miR-145, miR-155, and miR-382 in the exosomes from BC and noncancer patients, proving a correlation between the concentration of exosomes and the status of malignant breast tumors (46, 47). This phenomenon has been hotly discussed by many scholars. Anyway, the potential efficacy of exosomes to be a vital factor of microenvironment in TNBC diagnosis and treatment is always being researched.

With a deepening understanding of the mechanisms of exosomes, exosome-related gene expression status is associated with tumor progression. Therefore, three exosome-related genes were included in our exosome-related gene model by univariate Cox and LASSO Cox regression analysis. The prognosis of high-risk group and low-risk group patients were distinct different and patients in the low-risk group had a significantly higher survival ratio. Additionally, the ROC curve analysis confirmed that the established prognostic signature was powerful in predicting the OS for TNBC patients. Subsequently, the TNBC patients from the GEO cohort were categorized into high and low risk groups according to the median value of risk score and the group with lower risk score had a better prognosis rather than higher risk score. Hence, the three-gene signature along with covariates including race, age, and tumor stage were involved in the univariate and multivariate Cox regression models, proving that exosome-related gene risk score and tumor stage were independent prognostic factors for TNBC patients. Furthermore, R clusterProfiler package was applied to identify pathways enriched in the different risk groups. The result suggested that a majority of GO terms and KEGG pathways were related to lymphocyte activation and biological processes.

Immunologists discovered that exosomes play a vital role in antigen specific immune responses. Exosomes carry MHC-peptide complexes and antigens to increase the number of dendritic cells (DCs), which can present antigenic peptides to T cells (17). Notably, some studies indicated that exosomes released by tumors also bear various immunosuppressive molecules, for instance, CD4 and CD8 T lymphocytes (19, 21), NK cells (23, 24), regulatory T lymphocytes (25), and myeloid cells (26). Besides, some experiments *in vivo* mouse models obviously demonstrated that the antigen-shuttle function of exosomes overcame their inhibitory effects on immune cells in conditions of artificial overexpression of an antigen (24). By contrast, several groups favored the idea that, as for tumors, exosomes could inhibit anti-tumor immune responses and promote tumor progression such as migration and angiogenesis to form metastases (48). Recently, studies observed that patients with large malignant tumors had the increasing numbers of exosomes carrying tumor markers, which might only be the result of tumor expansion, instead of actively participating of vesicles in tumor progression of (49). Although, exosome secreted by tumors plays a vital role in immune system, the interaction mechanism is still much less explored.

Several immune activities are closely related to exosomes in tumors. Exosomes released by tumors can transfer antigens to DCs to activate specific T cells and make the activating ligands of NK cells and macrophages exposed to further promote immune responses (18). Oppositely, they carry different signals that can suppress various immune cells. Recently, a study about exosomal miRNA in breast cancer suggested that the transmission of miR-138-5p *via* exosomes could led to downregulation of KDM6B expression, inhibition of M1 polarization and stimulation of M2 polarization. Therefore, the relationship between exosomes and immune system was further detected (50). It has been reported that ESTIMATE scores could be used to predict survival time of patient with cancer to further clarify the facilitating effect of the microenvironment to tumor cells infiltration (51). In addition, stromal and immune cells from the TME play a crucial role in the tumorigenesis and tumor progression, related to the prognosis of patients with malignant tumors. In our study, low exosome-related risk group indicated a higher immune and ESTIMATE scores rather than high risk group. Subsequently, CIBERSORT algorithm was employed to calculate TIIC proportions and establish 22 kinds of TIIC profiles for high and low exosome-related risk groups. The result implicated that CD4^+^-activated memory T cells and M1 macrophages were both upregulated in low-risk group in patients from the TCGA and GEO databases, whereas M2 macrophages was downregulated in the low-risk group in patients from the TCGA and GEO databasses, respectively. As important regulators of the tumor microenvironment, exosomes have been suggested to play vital roles both in promoting immune response, as well as in inhibiting immune responses (52). Some studies implicated that exosome derived from tumor cell contained a lot of DNA, mRNAs, miRNAs, and enzymes, which shaped innate immune responses in tumor microenvironment. The exchange between immune cells and other cell types may be accomplished through the packaging of RNAs and DNAs (both single and double stranded) into exosomes that are selectively targeted and internalized with specific cell surface motifs. Fabbri et al. proposed that oncogenic genes miR-21 and miR-29a excreted by exosomes derived from lung cancer cells were able to combine TLR with mouse (TLR7) and human (TLR8), resulting in TLR-mediated NF-κB activation and secretion of the prometastatic inflammatory cytokines TNF-α and IL-6 (53). The mechanisms of tumor-immune system communication are of great significance for investigating the TIME regulatory factors. NK cells are known to kill tumor cells and produce cytotoxic cytokines, which can be trained through tumor-derived exosomes (54). Additionally, the status of DCs in tumor immune microenvironment (TIME) can also be influenced by exosomes released from tumor cells. Some other membrane and immune-related molecules involved in the recruitment and activation of immune cells in TIME were detected in exosomes derived from DCs (55). Moreover, exosomes secreted from tumor cells were demonstrated as one of stimuli to regulate macrophages. M2 was reported to be closely associated with the progression and prognosis of malignant tumor. Compared with M2, exosomes released by M1 could enhance antigen-specific cytotoxic T-cell responses to further enhance the activity of lipid calcium phosphate nanoparticle-encapsulated Trp2 vaccine. While, exosomes derived from M2 were verified to promote the growth and invasion of BC cells (56, 57). As for neutrophils, some cytokines and mediators were reported to be loaded *via* exosome-related neutrophils to modulate tumor progression. However, the stimuli in the TIME can in turn regulate the status of neutrophils to further shape tumor immune responses and influence tumor development (55). The accumulation of mast cells in tumor sites accounts for the construction of TIME. The regulator signals and other components released by mast cells were delivered to B cells, T cells, DCs, and tumor cells by exosomes (55). Exosomes also mediate the crosstalk between tumor cells and adaptive immune cells, which is one of major mediators for intercellular communications among adaptive immune cells, tumor cells. The dysfunction of T cell can be impaired because of tumor-derived exosomes. Some *in vitro* studies indicated that exosomes from tumor cells can induce the apoptosis of antigen-specific CD8^+^ T cells to suppress their functions (58). T-cell receptor and IL-2 receptor were also reported to be negatively modulated *via* tumor-related exosomes, resulting to the inhibition of T-cell proliferation (59). In addition to diverse categories of immune cells, other cellular constituents of the tumor microenvironment, involving mesenchymal stem cells, fibroblasts, and endothelial cells, play an aggressive role in tumor inception, promotion, proliferation, and metastasis. Tumor cell-derived exosome could educate normal MSCs with a protumor phenotype. Exosomes derived from MSCs with inflammatory cytokine stimulation include various mediators to suppress not only the progression of B cells, T cells, and NK cells but also the differentiation and antibody production of plasma cells, as well as to induce Tregs (60–62).

Immune checkpoints play a vital role in carcinogenesis by facilitating tumor immunosuppression. Stimulating the immune checkpoint targets can prevent tumors from attacking, including PD-L1, CTLA-4, LAG3, and TIM-3. The functions of these molecules are to inhibit T-cell receptor from activating downstream signals, thereby eliminating cytotoxic T lymphocytes and suppressing antitumor immunity (63). On the contrary, it was reported that PD-1 receptor and PD-L1 as a pair of T-cell immune response costimulatory molecules play a negative role in adoptive immunity by inhibiting T lymphocyte function. AiErken et al. reported that PD-L1 expression and tumor-infiltrating lymphocytes (TILs) were particularly biologically important in TNBC. The OS and DFS of patients with negative PD-L1 expression were shorter than PD-L1 expression (64). Notably, according to recent researches, TILs was able to become a basic marker in predicting treatment response (65). Additionally, previous studies reported that LAG3 positive intraepithelial tumor infiltrating lymphocytes (iTILs) were enriched in ER-negative breast tumors and considered an independent favorable prognostic factor, and the high expression of LAG3 in tissues was related to the good prognosis of triple negative breast cancer (66). In this research, PD-L1, LAG3, TIM-3, and CTLA-4 were upregulated in the low-risk group, as well. The high expression levels of PD-L1 might be associated with TIL-mediated antitumor inflammatory, indicating that cells in immune system are active (64). However, in some studies, the expression levels of CTLA-4 and TIM-3 were high in TNBC with poor clinical outcomes, which might be associated with the different TIL characters of different TNBC subtypes, supporting potential immune checkpoint blockade combination strategies to be a novel therapy for BC.

Neoadjuvant chemotherapy has been recognized to play better efficacy in BC patients without metastasis in lymph nodes, and therefore the indication for neoadjuvant chemotherapy were extended to patients with early TNBC. The patients in early-stage TNBC treated with neoadjuvant chemotherapy regimens based on paclitaxel and anthracyclines had a pathologic complete remission (PCR) rate of 30% to 40% (67), while those treated with carboplatin and paclitaxel had a PCR rate of 45% (68). Notably, patients treated with sequential dose-intensive combination of adriamycin, cyclophosphamide, paclitaxel, and carboplatin achieved a PCR rate in approximately 50% (69). The patients treated with chemotherapy regimen of docetaxel combined with carboplatin reached a PCR rate of 55% (70). Although the addition of carboplatin induces higher PCR rate, it also entails more complex adverse effects. Besides, targeted therapy and immunotherapy also play vital roles in TNBC treatment in recent years. Unfortunately, there are still no effective drugs for TNBC treatment, which is one of the reasons why TNBC has a high mortality rate. Eventually, we investigated whether the exosome-related risk model could predict chemosensitivity in TNBC. The results demonstrated that the IC_50_ values were statistically higher in the low-risk group for some anticancer agents. However, mithramycin, pipobroman, and sonidegib have seldom reported to apply in breast cancer treatment. It is the limitation of our study that lacking experiments to verify the effects of the three kinds of medicine on TNBC.

This study was the first one to establish and validate an exosome risk model according to three exosome-related genes, serving as an independent prognostic factor in TNBC patients. Our findings indicated that three-exosomal-gene risk model played a vital role in immune infiltration and has a close relationship with the prognosis of TNBC. Besides, some limitations of our study should be considered. A part of a clinical data in the TCGA or GEO cohort is incomplete, and the missing data may not be completely random, causing the bias in the clinical correlation analysis. In addition, the risk model was only established by exosome-related genes, but some other hot biomarker genes were absent. We only identified the association between the exosome-related risk model and immune infiltration, while the correlation between exosomes and TIME was seldom involved. Consequently, it requires a wider range of multicenter clinical verification to support our hypothesis and further experiments are needed to validate the association between exosomes and immune cells to give novel insights in the immunotherapy of TNBC patients.

## Conclusion

By combining bioinformatics tools and related algorithms, an exosome risk model that associated with immune infiltration was established and validated to predict the prognosis of TNBC patients. It can serve as an independent prognostic factor and bring new insights into the treatment of TNBC.

## Data Availability Statement

The datasets presented in this study can be found in online repositories. The names of the repository/repositories and accession number(s) can be found in the article/**Supplementary Material**.

## Author Contributions

Conception and design: PQ, QG, and JL. Development of methodology: QG and PQ. Acquisition of data (acquiring databases, data processing, etc.): PQ and QG. Analysis and interpretation of data (e.g., statistical analysis, biostatistics, computational analysis): PQ, QG, and JC. Writing, review, and/or revision of the manuscript: QG, QY, PQ, and JL. Administrative, technical, or material support: PQ, QG, and JL. Study supervision: JL. All authors contributed to the article and approved the submitted version.

## Conflict of Interest

The authors declare that the research was conducted in the absence of any commercial or financial relationships that could be construed as a potential conflict of interest.

## Publisher’s Note

All claims expressed in this article are solely those of the authors and do not necessarily represent those of their affiliated organizations, or those of the publisher, the editors and the reviewers. Any product that may be evaluated in this article, or claim that may be made by its manufacturer, is not guaranteed or endorsed by the publisher.

## References

[1] Siegel R Miller K Jemal A. Cancer Statistics, 2020. CA Cancer J Clin (2020) 70(1):7–30. doi: 10.3322/caac.21590 31912902

[2] Rastelli F Biancanelli S Falzetta A Martignetti A Casi C Bascioni R. Triple-Negative Breast Cancer: Current State of the Art. Tumori (2010) 96(6):875–88. doi: 10.1177/548.6505 21388048

[3] Harbeck N Gnant M. Breast Cancer. Lancet (London England) (2017) 389(10074):1134–50. doi: 10.1016/s0140-6736(16)31891-8 27865536

[4] Bergin A Loi S. Triple-Negative Breast Cancer: Recent Treatment Advances. F1000Research (2019) 8. doi: 10.12688/f1000research.18888.1 PMC6681627 31448088

[5] Gupta G Collier A Lee D Hoefer R Zheleva V Siewertsz van Reesema L. Perspectives on Triple-Negative Breast Cancer: Current Treatment Strategies, Unmet Needs, and Potential Targets for Future Therapies. Cancers (Basel) (2020) 12(9):2392. doi: 10.3390/cancers12092392 PMC7565566 32846967

[6] Tan D Marchió C Jones R Savage K Smith I Dowsett M. Triple Negative Breast Cancer: Molecular Profiling and Prognostic Impact in Adjuvant Anthracycline-Treated Patients. Breast Cancer Res Treat (2008) 111(1):27–44. doi: 10.1007/s10549-007-9756-8 17922188

[7] Akers J Gonda D Kim R Carter B Chen C. Biogenesis of Extracellular Vesicles (Ev): Exosomes, Microvesicles, Retrovirus-Like Vesicles, and Apoptotic Bodies. J Neurooncol (2013) 113(1):1–11. doi: 10.1007/s11060-013-1084-8 23456661 PMC5533094

[8] Piombino C Mastrolia I Omarini C Candini O Dominici M Piacentini F. The Role of Exosomes in Breast Cancer Diagnosis. Biomedicines (2021) 9(3):312. doi: 10.3390/biomedicines9030312 33803776 PMC8003248

[9] Pan B Johnstone R. Fate of the Transferrin Receptor During Maturation of Sheep Reticulocytes in Vitro: Selective Externalization of the Receptor. Cell (1983) 33(3):967–78. doi: 10.1016/0092-8674(83)90040-5 6307529

[10] Johnstone R. The Jeanne Manery-Fisher Memorial Lecture 1991. Maturation of Reticulocytes: Formation of Exosomes as a Mechanism for Shedding Membrane Proteins. Biochem Cell Biol = Biochimie Biologie Cellulaire (1992) 70:179–90. doi: 10.1139/o92-028 1515120

[11] Peinado H Alečković M Lavotshkin S Matei I Costa-Silva B Moreno-Bueno G. Melanoma Exosomes Educate Bone Marrow Progenitor Cells Toward a Pro-Metastatic Phenotype Through Met. Nat Med (2012) 18(6):883–91. doi: 10.1038/nm.2753 PMC3645291 22635005

[12] Yáñez-Mó M Siljander P Andreu Z Zavec A Borràs F Buzas E. Biological Properties of Extracellular Vesicles and Their Physiological Functions. J extracellular vesicles (2015) 4:27066. doi: 10.3402/jev.v4.27066 25979354 PMC4433489

[13] Adamczyk K Klein-Scory S Tehrani M Warnken U Schmiegel W Schnölzer M. Characterization of Soluble and Exosomal Forms of the Egfr Released From Pancreatic Cancer Cells. Life Sci (2011) 89:304–12. doi: 10.1016/j.lfs.2011.06.020 21763319

[14] Baran J Baj-Krzyworzeka M Weglarczyk K Szatanek R Zembala M Barbasz J. Circulating Tumour-Derived Microvesicles in Plasma of Gastric Cancer Patients. Cancer Immunol Immunother CII (2010) 59(6):841–50. doi: 10.1007/s00262-009-0808-2 PMC11030063 20043223

[15] Ciravolo V Huber V Ghedini G Venturelli E Bianchi F Campiglio M. Potential Role of Her2-Overexpressing Exosomes in Countering Trastuzumab-Based Therapy. J Cell Physiol (2012) 227(2):658–67. doi: 10.1002/jcp.22773 21465472

[16] Chaudhary P Gibbs L Maji S Lewis C Suzuki S Vishwanatha J. Serum Exosomal-Annexin A2 Is Associated With African-American Triple-Negative Breast Cancer and Promotes Angiogenesis. Breast Cancer Res BCR (2020) 22(1):11. doi: 10.1186/s13058-020-1251-8 31992335 PMC6986157

[17] Raposo G Nijman H Stoorvogel W Liejendekker R Harding C Melief C. B Lymphocytes Secrete Antigen-Presenting Vesicles. J Exp Med (1996) 183(3):1161–72. doi: 10.1084/jem.183.3.1161 PMC2192324 8642258

[18] Bobrie A Colombo M Raposo G Théry C. Exosome Secretion: Molecular Mechanisms and Roles in Immune Responses. Traffic (Copenhagen Denmark) (2011) 12(12):1659–68. doi: 10.1111/j.1600-0854.2011.01225.x 21645191

[19] Andreola G Rivoltini L Castelli C Huber V Perego P Deho P. Induction of Lymphocyte Apoptosis by Tumor Cell Secretion of Fasl-Bearing Microvesicles. J Exp Med (2002) 195(10):1303–16. doi: 10.1084/jem.20011624 PMC2193755 12021310

[20] Huber V Fais S Iero M Lugini L Canese P Squarcina P. Human Colorectal Cancer Cells Induce T-Cell Death Through Release of Proapoptotic Microvesicles: Role in Immune Escape. Gastroenterology (2005) 128(7):1796–804. doi: 10.1053/j.gastro.2005.03.045 15940614

[21] Taylor D Gerçel-Taylor C Lyons K Stanson J Whiteside T. T-Cell Apoptosis and Suppression of T-Cell Receptor/Cd3-Zeta by Fas Ligand-Containing Membrane Vesicles Shed From Ovarian Tumors. Clin Cancer Res an Off J Am Assoc Cancer Res (2003) 9(14):5113–9. 14613988

[22] Clayton A Mitchell J Court J Mason M and Tabi Z. Human Tumor-Derived Exosomes Selectively Impair Lymphocyte Responses to Interleukin-2. Cancer Res (2007) 67(15):7458–66. doi: 10.1158/0008-5472.can-06-3456 17671216

[23] Clayton A Mitchell J Court J Linnane S Mason M and Tabi Z. Human Tumor-Derived Exosomes Down-Modulate Nkg2d Expression. J Immunol (Baltimore Md 1950) (2008) 180(11):7249–58. doi: 10.4049/jimmunol.180.11.7249 18490724

[24] Liu C Yu S Zinn K Wang J Zhang L Jia Y. Murine Mammary Carcinoma Exosomes Promote Tumor Growth by Suppression of Nk Cell Function. J Immunol (Baltimore Md 1950) (2006) 176(3):1375–85. doi: 10.4049/jimmunol.176.3.1375 16424164

[25] Szajnik M Czystowska M Szczepanski M. Mandapathil M and Whiteside T. Tumor-Derived Microvesicles Induce, Expand and Up-Regulate Biological Activities of Human Regulatory T Cells (Treg). PloS One (2010) 5(7):e11469. doi: 10.1371/journal.pone.0011469 20661468 PMC2908536

[26] Valenti R Huber V Filipazzi P Pilla L Sovena G Villa A. Human Tumor-Released Microvesicles Promote the Differentiation of Myeloid Cells With Transforming Growth Factor-Beta-Mediated Suppressive Activity on T Lymphocytes. Cancer Res (2006) 66(18):9290–8. doi: 10.1158/0008-5472.can-06-1819 16982774

[27] Kennedy L. And Salama A. A Review of Cancer Immunotherapy Toxicity. CA Cancer J Clin (2020) 70(2):86–104. doi: 10.3322/caac.21596 31944278

[28] Hanahan D Coussens L. Accessories to the Crime: Functions of Cells Recruited to the Tumor Microenvironment. Cancer Cell (2012) 21(3):309–22. doi: 10.1016/j.ccr.2012.02.022 22439926

[29] Byrne A Savas P Sant S Li R Virassamy B Luen S. Tissue-Resident Memory T Cells in Breast Cancer Control and Immunotherapy Responses. Nat Rev Clin Oncol (2020) 17(6):341–8. doi: 10.1038/s41571-020-0333-y 32112054

[30] Savas P Salgado R Denkert C Sotiriou C Darcy P Smyth M. Clinical Relevance of Host Immunity in Breast Cancer: From Tils to the Clinic. Nat Rev Clin Oncol (2016) 13(4):228–41. doi: 10.1038/nrclinonc.2015.215 26667975

[31] Sanchez K Page D McArthur H. Immunotherapy in Breast Cancer: An Overview of Modern Checkpoint Blockade Strategies and Vaccines. Curr Probl Cancer (2016) 40:151–62. doi: 10.1016/j.currproblcancer.2016.09.009 27855963

[32] Schmid P Adams S Rugo H Schneeweiss A Barrios C Iwata H. Atezolizumab and Nab-Paclitaxel in Advanced Triple-Negative Breast Cancer. N Engl J Med (2018) 379(22):2108–21. doi: 10.1056/NEJMoa1809615 30345906

[33] Zuo Z Xiong J Zeng C Jiang Y Xiong K Tao H. Exploration of a Robust and Prognostic Immune Related Gene Signature for Cervical Squamous Cell Carcinoma. Front Mol Biosci (2021) 8:625470. doi: 10.3389/fmolb.2021.625470 33748188 PMC7967036

[34] Kim J Lee E Park K Park W Jung H Ahn J. Immune Signature of Metastatic Breast Cancer: Identifying Predictive Markers of Immunotherapy Response. Oncotarget (2017) 8(29):47400–11. doi: 10.18632/oncotarget.17653 PMC5564574 28537889

[35] Saleh R Toor S Khalaf S Elkord E. Breast Cancer Cells and Pd-1/Pd-L1 Blockade Upregulate the Expression of Pd-1, Ctla-4, Tim-3 and Lag-3 Immune Checkpoints in Cd4 T Cells. Vaccines (2019) 7(4):149. doi: 10.3390/vaccines7040149 PMC6963740 31614877

[36] Wang H Lengerich B Aragam B Xing E. Precision Lasso: Accounting for Correlations and Linear Dependencies in High-Dimensional Genomic Data. Bioinf (Oxford England) (2019) 35(7):1181–7. doi: 10.1093/bioinformatics/bty750 PMC6449749 30184048

[37] Yoshihara K Shahmoradgoli M Martínez E Vegesna R Kim H Torres-Garcia W. Inferring Tumour Purity and Stromal and Immune Cell Admixture From Expression Data. Nat Commun (2013) 4:2612. doi: 10.1038/ncomms3612 24113773 PMC3826632

[38] Newman A Liu C Green M Gentles A Feng W Xu Y. Robust Enumeration of Cell Subsets From Tissue Expression Profiles. Nat Methods (2015) 12(5):453–7. doi: 10.1038/nmeth.3337 PMC4739640 25822800

[39] Sun Y Haglund T Rogers A Ghanim A Sethu P. Review: Microfluidics Technologies for Blood-Based Cancer Liquid Biopsies. Anal Chim Acta (2018) 1012:10–29. doi: 10.1016/j.aca.2017.12.050 29475470

[40] Costa-Silva B Aiello N Ocean A Singh S Zhang H Thakur B. Pancreatic Cancer Exosomes Initiate Pre-Metastatic Niche Formation in the Liver. Nat Cell Biol (2015) 17(6):816–26. doi: 10.1038/ncb3169 PMC5769922 25985394

[41] Jayaseelan V. Emerging Role of Exosomes as Promising Diagnostic Tool for Cancer. Cancer Gene Ther (2020) 27(6):395–8. doi: 10.1038/s41417-019-0136-4 31477807

[42] McKiernan J Donovan M O’Neill V Bentink S Noerholm M Belzer S. A Novel Urine Exosome Gene Expression Assay to Predict High-Grade Prostate Cancer at Initial Biopsy. JAMA Oncol (2016) 2(7):882–9. doi: 10.1001/jamaoncol.2016.0097 27032035

[43] Wang X Zhong W Bu J Li Y Li R Nie R. Exosomal Protein Cd82 as a Diagnostic Biomarker for Precision Medicine for Breast Cancer. Mol Carcinog (2019) 58(5):674–85. doi: 10.1002/mc.22960 30604894

[44] Kibria G Ramos E Lee K Bedoyan S Huang S Samaeekia R. A Rapid, Automated Surface Protein Profiling of Single Circulating Exosomes in Human Blood. Sci Rep (2016) 6:36502. doi: 10.1038/srep36502 27819324 PMC5098148

[45] Chao M Jaiswal S Weissman-Tsukamoto R Alizadeh A Gentles A Volkmer J. Calreticulin Is the Dominant Pro-Phagocytic Signal on Multiple Human Cancers and Is Counterbalanced by Cd47. Sci Transl Med (2010) 2(63):63ra94. doi: 10.1126/scitranslmed.3001375 PMC4126904 21178137

[46] Mar-Aguilar F Mendoza-Ramírez J Malagón-Santiago I Espino-Silva P Santuario-Facio S Ruiz-Flores P. Serum Circulating Microrna Profiling for Identification of Potential Breast Cancer Biomarkers. Dis Markers (2013) 34(3):163–9. doi: 10.3233/dma-120957 PMC3810231 23334650

[47] Gonzalez-Villasana V Rashed M Gonzalez-Cantú Y Bayraktar R Menchaca-Arredondo J Vazquez-Guillen J. Presence of Circulating Mir-145, Mir-155, and Mir-382 in Exosomes Isolated From Serum of Breast Cancer Patients and Healthy Donors. Dis Markers (2019) 2019:6852917. doi: 10.1155/2019/6852917 30891102 PMC6390256

[48] Ichim T Zhong Z Kaushal S Zheng X Ren X Hao X. Exosomes as a Tumor Immune Escape Mechanism: Possible Therapeutic Implications. J Transl Med (2008) 6:37. doi: 10.1186/1479-5876-6-37 18644158 PMC2504474

[49] Taylor D Gercel-Taylor C. Microrna Signatures of Tumor-Derived Exosomes as Diagnostic Biomarkers of Ovarian Cancer. Gynecol Oncol (2008) 110(1):13–21. doi: 10.1016/j.ygyno.2008.04.033 18589210

[50] Xun J Du L Gao R Shen L Wang D Kang L. Cancer-Derived Exosomal Mir-138-5p Modulates Polarization of Tumor-Associated Macrophages Through Inhibition of Kdm6b. Theranostics (2021) 11(14):6847–59. doi: 10.7150/thno.51864 PMC8171095 34093857

[51] Motakis E Ivshina A Kuznetsov V. Data-Driven Approach to Predict Survival of Cancer Patients: Estimation of Microarray Genes’ Prediction Significance by Cox Proportional Hazard Regression Model. IEEE Eng Med Biol magazine Q magazine Eng Med Biol Soc (2009) 28(4):58–66. doi: 10.1109/memb.2009.932937 19622426

[52] Greening D Gopal S Xu R Simpson R Chen W. Exosomes and Their Roles in Immune Regulation and Cancer. Semin Cell Dev Biol (2015) 40:72–81. doi: 10.1016/j.semcdb.2015.02.009 25724562

[53] Fabbri M Paone A Calore F Galli R Gaudio E Santhanam R. Micrornas Bind to Toll-Like Receptors to Induce Prometastatic Inflammatory Response. Proc Natl Acad Sci USA (2012) 109(31):E2110–6. doi: 10.1073/pnas.1209414109 PMC3412003 22753494

[54] Morvan M Lanier L. Nk Cells and Cancer: You Can Teach Innate Cells New Tricks. Nat Rev Cancer (2016) 16(1):7–19. doi: 10.1038/nrc.2015.5 26694935

[55] Huang Y Liu K Li Q Yao Y Wang Y. Exosomes Function in Tumor Immune Microenvironment. Adv Exp Med Biol (2018) 1056:109–22. doi: 10.1007/978-3-319-74470-4_7 29754177

[56] Cheng L Wang Y Huang L. Exosomes From M1-Polarized Macrophages Potentiate the Cancer Vaccine by Creating a Pro-Inflammatory Microenvironment in the Lymph Node. Mol Ther J Am Soc Gene Ther (2017) 25(7):1665–75. doi: 10.1016/j.ymthe.2017.02.007 PMC5498801 28284981

[57] Yang M Chen J Su F Yu B Su F Lin L. Microvesicles Secreted by Macrophages Shuttle Invasion-Potentiating Micrornas Into Breast Cancer Cells. Mol Cancer (2011) 10:117. doi: 10.1186/1476-4598-10-117 21939504 PMC3190352

[58] Wieckowski E Visus C Szajnik M Szczepanski M Storkus W Whiteside T. Tumor-Derived Microvesicles Promote Regulatory T Cell Expansion and Induce Apoptosis in Tumor-Reactive Activated Cd8+ T Lymphocytes. J Immunol (Baltimore Md 1950) (2009) 183(6):3720–30. doi: 10.4049/jimmunol.0900970 PMC3721354 19692638

[59] Whiteside T. Immune Modulation of T-Cell and Nk (Natural Killer) Cell Activities by Texs (Tumour-Derived Exosomes). Biochem Soc Trans (2013) 41(1):245–51. doi: 10.1042/bst20120265 PMC3721347 23356291

[60] Conforti A Scarsella M Starc N Giorda E Biagini S Proia A. Microvescicles Derived From Mesenchymal Stromal Cells Are Not as Effective as Their Cellular Counterpart in the Ability to Modulate Immune Responses in Vitro. Stem Cells Dev (2014) 23(21):2591–9. doi: 10.1089/scd.2014.0091 PMC4201301 24937591

[61] Di Trapani M Bassi G Midolo M Gatti A Kamga P Cassaro A. Differential and Transferable Modulatory Effects of Mesenchymal Stromal Cell-Derived Extracellular Vesicles on T, B and Nk Cell Functions. Sci Rep (2016) 6:24120. doi: 10.1038/srep24120 27071676 PMC4829861

[62] Del Fattore A Luciano R Pascucci L Goffredo B Giorda E Scapaticci M. Immunoregulatory Effects of Mesenchymal Stem Cell-Derived Extracellular Vesicles on T Lymphocytes. Cell Transplant (2015) 24(12):2615–27. doi: 10.3727/096368915x687543 25695896

[63] Yi H Li Y Tan Y Fu S Tang F Deng X. Immune Checkpoint Inhibition for Triple-Negative Breast Cancer: Current Landscape and Future Perspectives. Front Oncol (2021) 11:648139. doi: 10.3389/fonc.2021.648139 34094935 PMC8170306

[64] AiErken N Shi H Zhou Y Shao N Zhang J Shi Y. High Pd-L1 Expression Is Closely Associated With Tumor-Infiltrating Lymphocytes and Leads to Good Clinical Outcomes in Chinese Triple Negative Breast Cancer Patients. Int J Biol Sci (2017) 13(9):1172–9. doi: 10.7150/ijbs.20868 PMC5666332 29104508

[65] Tumeh P Harview C Yearley J Shintaku I Taylor E Robert L. Pd-1 Blockade Induces Responses by Inhibiting Adaptive Immune Resistance. Nature (2014) 515(7528):568–71. doi: 10.1038/nature13954 PMC4246418 25428505

[66] Burugu S Gao D Leung S Chia S Nielsen T. Lag-3+ Tumor Infiltrating Lymphocytes in Breast Cancer: Clinical Correlates and Association With Pd-1/Pd-L1+ Tumors. Ann Oncol Off J Eur Soc Med Oncol (2017) 28(12):2977–84. doi: 10.1093/annonc/mdx557 29045526

[67] Gianni L Mansutti M Anton A Calvo L Bisagni G Bermejo B. Comparing Neoadjuvant Nab-Paclitaxel Vs Paclitaxel Both Followed by Anthracycline Regimens in Women With Erbb2/Her2-Negative Breast Cancer-The Evaluating Treatment With Neoadjuvant Abraxane (Etna) Trial: A Randomized Phase 3 Clinical Trial. JAMA Oncol (2018) 4(3):302–8. doi: 10.1001/jamaoncol.2017.4612 PMC5885830 29327055

[68] Loibl S O’Shaughnessy J Untch M Sikov W Rugo H McKee M. Addition of the Parp Inhibitor Veliparib Plus Carboplatin or Carboplatin Alone to Standard Neoadjuvant Chemotherapy in Triple-Negative Breast Cancer (Brightness): A Randomised, Phase 3 Trial. Lancet Oncol (2018) 19(4):497–509. doi: 10.1016/s1470-2045(18)30111-6 29501363

[69] Dieci M Del Mastro L Cinquini M Montemurro F Biganzoli L Cortesi L. Inclusion of Platinum Agents in Neoadjuvant Chemotherapy Regimens for Triple-Negative Breast Cancer Patients: Development of Grade (Grades of Recommendation, Assessment, Development and Evaluation) Recommendation by the Italian Association of Medical Oncology (Aiom). Cancers (Basel) (2019) 11(8):1137. doi: 10.3390/cancers11081137 PMC6721549 31398896

[70] Echavarria I López-Tarruella S Picornell A García-Saenz J Jerez Y Hoadley K. Pathological Response in a Triple-Negative Breast Cancer Cohort Treated With Neoadjuvant Carboplatin and Docetaxel According to Lehmann’s Refined Classification. Clin Cancer Res an Off J Am Assoc Cancer Res (2018) 24(8):1845–52. doi: 10.1158/1078-0432.ccr-17-1912 PMC5899625 29378733

